# A Rare Case of Psychogenic Nonepileptic Seizure Following Transcranial Magnetic Stimulation

**DOI:** 10.7759/cureus.82845

**Published:** 2025-04-23

**Authors:** Nicole Muravsky, Raymond Zhang, Kathleen Z He, Franklin E Caldera, Yejia Zhang

**Affiliations:** 1 Physical Medicine and Rehabilitation, University of Pennsylvania, Philadelphia, USA; 2 Rehabilitation Medicine, Corporal Michael J. Crescenz Veterans Affairs Medical Center, Philadelphia, USA; 3 Physical Medicine and Rehabilitation, University of Massachusetts Amherst, Amherst, USA; 4 Physical Medicine and Rehabilitation, Hospital of the University of Pennsylvania, Philadelphia, USA

**Keywords:** acupuncture treatment, depression, psychogenic nonepileptic seizure, side-effects, transcranial magnetic stimulation therapy

## Abstract

Psychogenic nonepileptic seizures (PNES), also referred to as functional seizures, are events that mimic epileptic seizures but are not triggered by abnormal electrical activity in the brain. According to the International Statistical Classification of Diseases (ICD)-11, PNES are classified as dissociative disorders. Transcranial magnetic stimulation (TMS) is a non-invasive brain stimulation therapy commonly used to treat major depression, particularly in cases where other treatments have not been effective. PNES has not been associated with TMS previously. Here, we describe a 52-year-old Caucasian male who presented to the acupuncture clinic with multiple episodes of sudden loss of consciousness following TMS for a long history of major depression. The episodes of unconsciousness occurred up to five times per day. During an electroencephalograph (EEG) session, the patient had an episode that included poor balance, "shaking," head nodding, and a robotic/slowed voice, although no epileptic activity was captured on EEG. His illness was therefore diagnosed as PNES activity. He underwent treatment with body acupuncture and auricular acupressure and improved, with reduced number and duration of episodes. PNES following TMS has not been reported previously. A strong magnetic field can potentially disrupt normal neurotransmission and neuronal metabolism, resulting in PNES. The beneficial effects of acupuncture have been documented, but the mechanism of action has not been elucidated.

## Introduction

The estimated annual incidence of psychogenic nonepileptic seizures (PNES) is up to 4.90 cases per 100,000 individuals. These episodes can occur across diverse racial and cultural groups, most frequently emerging in individuals in their third decade of life. PNES episodes typically take place in the presence of witnesses and can appear in various forms. However, the majority fall into two main categories: “convulsive” and “swoon” types. Convulsive PNES can resemble tonic-clonic seizures, involving a sudden fall if the person is standing, with rhythmic movements of all four limbs, and often the trunk and head. In contrast, swoon-type PNES can be mistaken for a fainting episode (syncope), as it involves a collapse to the ground or a slump to the side when seated, with minimal or no movement. Some episodes may show features of both types and are considered intermediate. The primary condition to distinguish from PNES is epileptic seizures, although swoon-type PNES must also be differentiated from syncope, whether vasovagal or cardiac in origin. The most reliable method for confirming a diagnosis involves video electroencephalograph (EEG) monitoring with ECG, capturing episodes that are verified by witnesses as representative of typical events [1].

Functional seizures are believed to primarily stem from psychological factors, although structural changes have been observed through magnetic resonance imaging (MRI) [2]. One theory suggests that neuroinflammation - an inflammatory response within the nervous system - may play a role in their development [3]. Communicating the diagnosis to patients is a crucial part of management, with a notable proportion - ranging from 17% to 40% - experiencing a cessation of episodes after being informed of the diagnosis. Ongoing care typically involves continued neurological oversight, gradual discontinuation of antiseizure medications, as well as neuropsychological assessments and psychiatric evaluations. Psychotherapy, provided by a psychologist or psychiatrist, remains the cornerstone of treatment [1].

Transcranial magnetic stimulation (TMS) treatment involves placing an electromagnetic coil on the scalp and delivering a series of short magnetic pulses to the dorsolateral prefrontal cortex, an area of the brain that modulates affective processing [4]. TMS for treatment of depression, typically targeting the dorsolateral prefrontal cortex, has a response rate ranging from 29% to 46% [5]. Common side effects include headache, a low incidence of seizures, and induced hypomania. PNES has not been reported previously as a side-effect of TMS [6]. Acupuncture is an ancient practice of traditional Chinese medicine that is believed to modulate the nervous system, possibly by normalizing cell-cell communications along the Meridian, pathways connecting the organ systems. Acupuncture treatments have been used as a low-risk modality to treat pain and mental issues with varying degrees of success. This patient was referred for acupuncture after failed conventional treatments, including medications and psychotherapy. Following treatments with both needle acupuncture and auricular acupressure, the patient had fewer PNES episodes.

## Case presentation

The patient was a pleasant 52-year-old Caucasian man with a past medical history of mental health issues, including major depression and generalized anxiety disorder, as well as other comorbidities, such as hypertension, diabetes, obesity, hyperlipidemia, cervical myelopathy, osteopenia, obstructive sleep apnea, and lower back and neck pain and limb movement disorder. After starting TMS treatments five years ago for depression, he presented with falling, associated with brief loss of consciousness, poor balance, and limb "shaking," without resulting in significant injuries. 

He has no past history or family history of conversion symptoms or PNES. Confounding factors include stress; his neighbor smokes cigarettes, and his wife's illness has been mentioned as a stress factor. Patient was on Duloxetine 60 mg, Lamotrigine 200 mg, and Trazodone 150 mg once daily. He tried Buspirone 10 mg twice a day but found it unhelpful and has been discontinued. He was referred to the acupuncture clinic as a last resort because he did not improve with medication and psychotherapy.

Physical examination revealed that he was mildly overweight and that speech and cognition were normal. Neurological examination, including cranial and limb sensation and reflexes, was unremarkable.

**Figure 1:**
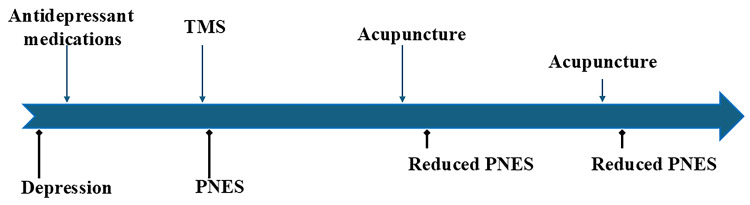
Timeline of symptoms and treatments Types of treatment are shown by vertical blue arrows above the timeline; symptoms are denoted by black arrows with diamond heads under the timeline. The frequency of the various events (treatment or symptoms) is illustrated semi-quantitatively by the lengths of the vertical arrows. PNES: psychogenic nonepileptic seizure; TMS: transcranial magnetic stimulation

He underwent auriculotherapy acupressure treatment with Vaccaria seeds placed at the patient’s Gate of Spirit (triangular fossa in the external ear), thalamus (middle of the base of the antitragus), and tragus points. The patient also underwent needle acupuncture at the bilateral Hegu (between the base of the thumb and index fingers), Zusanli (below the knee and lateral to the tibia), Shousanli (on the outer surface of the forearm, below the elbow crease), Yintang (at the midpoint between the eyebrows), and Baihui (on the highest place of the head). The patient was instructed to use a transcutaneous electrical nerve stimulation (TENS) unit to stimulate Zusanli at home. The patient improved after several months of treatment, with a decreasing number and shorter duration of episodes (<1 episode/day, lasting <1 minute). However, the patient returned to the acupuncture clinic one year later because his PNES episodes increased to five times per day, each lasting a few minutes. He attributed this recurrence to social and family stresses. He resumed acupressure and acupuncture treatments every three months, which helped reduce the frequency and duration of these episodes. The patient also had brief speech therapy and regular psychological counseling. He has had rare PNES episodes since then, and the symptoms are not as debilitating as he presented initially (Figure 1).

## Discussion

We have, for the first time, captured a PNES episode during an EEG study of a patient who developed symptoms following TMS treatment. Because the episodes included poor balance, "shaking," head nodding, and a robotic/slowed voice, this patient can be best classified as an intermediate type PNES. The primary condition to distinguish from PNES is epileptic seizures; lack of epileptic activity during a witnessed episode ruled this out. Although conversion disorder remained a differential diagnosis, the patient’s awareness of his episodes and willingness to receive treatments make conversion disorder less likely.

Although it is difficult to show the effects of electromagnetic energy, recent work with sophisticated instruments has shown diminished cellular autofluorescence with an external magnetic field. This experiment directly linked magnetic field effects on chemical reactions measured in solution and chemical reactions taking place in living cells [7].

Auricular acupuncture has been shown to reduce PNES event frequency in some patients [8], but the mechanism of action is unclear. Sensory innervation to the external ear is supplied by both cranial and spinal nerves, and stimulating these nerves may modulate cerebral cortex activity. Acupuncture is increasingly used as an alternative treatment for neuropsychological issues, with only empirical evidence of benefit so far. Further investigation into cellular/molecular mechanisms of action is clearly warranted.

## Conclusions

We have described a rare case of PNES following TMS for the treatment of depression. Advancements in cell and molecular biology techniques now enable researchers to gain a deeper understanding of how magnetic waves influence living organisms. This progress has unveiled potential effects on cellular processes such as gene expression, signal transduction pathways, and cell behavior, which were previously challenging to study in detail. Since brain tissues have limited regeneration potential, they may be more susceptible to damage. The case presented here raised concerns about the possible adverse effects of exposure to magnetic fields. Finally, although the patient improved with acupuncture treatments, the mechanism of action remains to be determined.
